# Factors associated with prolonged hospital stay in patients with autoimmune bullous diseases treated at an in-patient dermatology service

**DOI:** 10.1093/skinhd/vzag066

**Published:** 2026-06-09

**Authors:** Pedro Juan Saldarriaga-Muñoz, Valentina Rodelo-Sánchez, Isabela Agudelo-Aguilar, David Osorio-Álvarez, Sebastián Valencia-Montoya, Sebastián Bedoya-Mejía, Susana Chiquito-García, Geovanna Andrea Ayala-Monroy

**Affiliations:** Dermatology Service, Universidad CES, Medellín, Colombia; Dermatology Service, Universidad CES, Medellín, Colombia; Dermatology Service, Universidad CES, Medellín, Colombia; Plastic Surgery Service, Clínica CES, Medellín, Colombia; Universidad CES, Medellín, Colombia; Universidad CES, Medellín, Colombia; Dermatology Service, Universidad CES, Medellín, Colombia; Dermatology Service, Clínica CES, Medellín, Colombia; Dermatology Service, Universidad CES, Medellín, Colombia; Dermatology Service, Clínica CES, Medellín, Colombia

## Abstract

**Background:**

Bullous diseases carry a great burden and are often linked to high healthcare costs. Patients with ­bullous diseases are more vulnerable to infections and organ damage due to an incompetent skin barrier.

**Objectives:**

To determine the demographic, clinical and laboratory factors associated with prolonged hospital stay in patients with autoimmune bullous diseases.

**Methods:**

This was a cross-sectional study, with a follow-up cohort, including all dermatology consultations in a university hospital between 2017 and 2022 to identify patients with autoimmune bullous disease. Duration of stay and other variables were obtained from the patients’ medical records.

**Results:**

A total of 83 patients were included in the study, of whom the majority  had bullous pemphigoid (*n* = 45; 54%). There was significant metabolic comorbidity with hypertension (*n* = 51; 61%), diabetes (*n* = 31; 37%) and dyslipidaemia (*n* = 31; 37%). Fifty-eight per cent of patients had a prolonged hospital stay (>10 days). Among the associated variables, mucosal involvement, eosinophilia, in-hospital antibiotics and the absence of dipetidyl peptidase-4 (DPP4) inhibitor use stood out.

**Conclusions:**

Most patients with bullous diseases have prolonged hospital stays. The evaluation of mucous membranes, blood count and a basic chemical panel can help identify patients at greater risk of prolonged hospital stays. In addition, the use of DPP4 inhibitors and their rapid discontinuation could affect this outcome.

## Teaser

Cross-sectional study, with a follow-up cohort, including all dermatology consultations in a university hospital between 2017 and 2022 to identify patients with autoimmune bullous disease and factors associated with prolonged length of stay. Most patients with bullous diseases have prolonged hospital stays. The evaluation of mucous membranes, the blood count and the basic chemical panel can help identify those patients at greater risk of prolonged stays.

What is already known about this topic?

- Bullous diseases have a great disease burden and are often linked with high healthcare costs.
- These patients are more vulnerable to infections and organ damage due to skin barrier damage.

What does this study add?

- A thorough evaluation of comorbidities and medical treatments can identify factors associated with prolonged hospital stay.
- Clinical and laboratory analysis may also contribute to the identification of patients at risk of having a prolonged hospital stay.

Autoimmune bullous diseases (AIBDs) are characterized by the development of flaccid or tense blisters depending on the level of immune involvement in the epidermis or dermoepidermal junction.^1^ Although their presentation is varied, they commonly affect the adult population, especially those over 50 years of age, with various comorbidities including hypertension, diabetes and neuropsychiatric diseases.^1,2^

In the USA, the prevalence of hospitalizations associated with just one of these diseases, bullous pemphigoid (BP), increased from 25.84 to 32.60 cases per million between 2002 and 2012.^3^ Ren *et al*. have described an average hospital stay of 7.3 days in the same period.^3^ This goes hand in hand with a higher number of infections and chronic comorbidities, as well as an average ­in-hospital mortality rate of 2.9%, raising the costs of care.^3,4^

Although they are diseases associated with a considerable disease burden, there is no extensive information on the factors associated with prolonged hospital stay. This is important as a prolonged hospital stay carries with it an increased risk of infectious and thrombotic complications, among others, in addition to an increase in the costs associated with the disease.^4^ On the contrary, most studies have focused on identifying factors associated with mortality.^5,6^ This is especially true in the Latin American context, where most studies have focused on the characterization of patients with these diseases without analysing associated clinical outcomes.^7^

As such, the objective of this study was to determine the demographic, clinical and laboratory factors associated with prolonged hospital stay in patients with AIBDs treated in an in-patient dermatological institution in Medellín, Colombia, from January 2017 to December 2022.

## Patients and methods

This cross-sectional study followed a cohort of patients treated between 1 January 2017 and 31 December 2022 at an institution with an in-patient dermatology service that treats an average of 700 patients annually and has more than 40 years of experience. The medical records of all patients aged >18 years with bullous diseases seen during this period were evaluated, excluding pregnant patients and patients referred to other institutions during hospitalization. More than 3000 patients seen at the dermatology service over the course of the 5-year study period were considered potentially eligible for inclusion. Each patient was evaluated from the day of admission to hospital until their discharge from the institution.

The primary outcome was the duration of hospital stay (days) from admission until discharge. Based on a literature review, a duration of >10 days was used as the cutoff point for a prolonged hospital stay.^8–10^ We recorded patient variables such as demographic factors, clinical history and comorbidities; clinical findings such as affected body surface area and mucosal involvement; pathology findings such as blister level and infiltrate density; and laboratory findings such as blood count, kidney function and liver enzymes. These variables were considered as each of these may play a role in establishing greater prior frailty, more extensive disease involvement, greater inflammatory status and associated organ damage, all factors that can lead to longer hospital stays.

Data collection was carried out by four of the researchers; a pilot trial was carried out with the first 10 patients to standardize this process and avoid information biases. Demographic, clinical and laboratory data were taken directly from clinical records, while the pathology data were obtained from official reports issued by the dermatopathology service to avoid biases caused by the interpretation of findings by the treating dermatologist. Vital signs were obtained from nursing records, and laboratory reports directly from the internal record included in the medical record service.

For the qualitative variables, frequency tables were used, and their confidence intervals (CIs) were accompanied by a proportion. For quantitative variables, assumption of normality was verified by the Shapiro–Wilk statistical test, and measures of central tendency and dispersion are shown as appropriate. Likewise, for the bivariate analysis, a χ^2^ statistical test of independence and Fisher’s exact test were used. Crude prevalence ratios were calculated accompanied by their CI as an epidemiological measure. Binomial regression with log link was performed, showing adjusted prevalence ratios, as well as all those that met the Hosmer and Lemeshow criteria, which were statistically significant (*P* < 0.05), and were plausible. An interdependence analysis was carried out to identify multiple correspondence to create the profile of patients with prolonged and nonprolonged stays.

## Results

### Demographics

A total of 3000 records were evaluated from which 83 patients with AIBDs were identified, with a 2.8% prevalence. Of the 83 patients, 51 (61%) were women. The median age was 75 years (interquartile range 61–82). The most common diagnosis was BP (*n* = 45; 54%). Thirty-one per cent (*n* = 26) had recently started medications, of which the use of dipetidyl peptidase-4 (DPP4) inhibitors, diuretics and antibiotics stood out. The most frequent comorbidities were hypertension (*n* = 51; 61%), diabetes (*n* = 31; 37%) and dyslipidaemia (*n* = 31; 37%). There was oral mucosal involvement in 28% (*n* = 23) and scalp involvement in 25% (*n* = 21) of patients. Records showed leucocytosis in 70% (*n* = 58), anaemia in 33% (*n* = 27), eosinophilia in 33% (*n* = 27), hyperglycaemia in 31% (*n* = 26) and elevated creatinine in 30% (*n* = 25) (Tables 1, 2).

**Table 1:** Qualitative characteristics of 83 patients with bullous diseases who were treated in hospital

Variable	*n* (%)	95% CI
Clinical variables		
Sex		
Female	51 (61)	43–72
Male	32 (39)	28–49
Recent use of prescription drugs		
Recent use of prescription drugs	26 (31)	21–41
DPP4 inhibitors	10 (12)	5–19
Diuretics	10 (12)	5–19
Antibiotics	14 (17)	9–25
Anticonvulsive	3 (4)	0–8
NSAID	3 (4)	0–8
Opioids	2 (2)	0–6
Medical history		
Neoplasia	5 (6)	1–11
Haematological malignancy	2 (2)	0–6
Solid organ malignancy	4 (5)	0–9
Recent infection	16 (19)	11–28
Chronic hypertension	51 (61)	51–72
Diabetes mellitus	31 (37)	27–48
Hypothyroidism	11 (13)	6–21
Dyslipidaemia	31 (37)	27–48
Obesity	24 (29)	19–39
Neurological disease	24 (29)	19–39
Psychiatric disease	20 (24)	15–33
Autoimmune disease	5 (6)	1–11
Type of bullous disease		
Bullous pemphigoid	45 (54)	44–65
Foliaceus pemphigus	13 (16)	8–24
Vulgar pemphigus	19 (23)	14–32
IgA pemphigus	2 (2)	0–6
Acquired epidermodysplasia bullosa	1 (1)	0–4
Herpetiform dermatitis	0 (0)	0–0
Linear IgA dermatosis	2 (2)	0–6
Bullosis diabeticorum	1 (1)	0–4
Bullous lupus erythematosus	0 (0)	0–0
Special area involvement		
Scalp	21 (25)	16–35
Oral mucosa	23 (28)	18–37
Conjunctiva	3 (4)	0–7.6
Genital mucosa	6 (7)	2–13
Laboratory variables		
Anaemia		
No anaemia	55 (66)	56–76
Anaemia	27 (33)	23–43
No information	1 (1)	1–4
Leucocyte count		
Leucopenia	4 (5)	0–9
Normal range	20 (24)	15–33
Leucocytosis	58 (70)	60–80
No information	1 (1)	1–4
Eosinophil count		
Normal range	49 (59)	49–70
Eosinophilia	27 (33)	23–43
No information	7 (8)	3–14
Creatinine		
Normal range	56 (67)	57–78
Elevated	25 (30)	20–40
No information	2 (2)	1–6
Treatment variables		
Topical steroids		
High potency	34 (41)	30–52
Medium potency	38 (46)	35–57
Low potency	2 (2)	0–6
Not prescribed	9 (11)	2–13
Systemic steroids		
0–10 mg	2 (2)	0–6
>10–20 mg	5 (6)	1–11
>20–50 mg	49 (59)	49–70
>50 mg	22 (27)	17–36
Not prescribed	5 (6)	1–11
Opioids		
No	67 (81)	69–87
Yes	16 (19)	11–28
Antibiotics		
No	56 (67)	55–75
Yes	27 (33)	23–43
Duration of hospital stay		
Not prolonged	35 (42)	32–53
Prolonged	48 (58)	47–69

CI, confidence interval; DPP4, dipeptidyl peptidase-4; NSAID, nonsteroidal anti-inflammatory drug.

**Table 2:** Quantitative characteristics of 83 patients with bullous diseases treated in hospital

Variable	Median (IQR) or mean (SD)	Minimum	Maximum
Hospital stay (days)	11.0 (7.0–18.0)	2.0	54.0
Age (years)	75.0 (61.0–85.0)	18.0	102.0
Haemoglobin (mg dL^−1^)	12.9 (2.23)	8.00	16.90
Leucocyte count (mm^3^)	9914 (3327)	1015.0	17 310.0
Platelet count (G L^−1^)	283.0 (235.0–370.0)	31.8	818.0
Eosinophil count (mm^3^)	260.0 (50.0–670.0)	0.0	6420.0
C-reactive protein (mg dL^−1^)	2.00 (0.45–3.92)	0.05	19.50
Alanine transferase (U L^−1^)	23.0 (21.8)	4.30	136.50
Aspartate transferase (U L^−1^)	19.4 (10.0)	7.8	60.6
Gamma glutaryl transferase (U L^−1^)	26.5 (15.0–40.0)	11.0	289.0
Alkaline phosphatase (U/L^−1^)	96.2 (54.0)	40.0	343.0
Urea (mg dL^−1^)	26.00 (19.60–41.80)	8.00	124.10
Creatinine (mg dL^−1^)	0.89 (0.70–1.08)	0.30	3.30
Lactate dehydrogenase (U L^−1^)	190.5 (164.0–260.5)	97.0	693.0
Glycaemia (mg dL^−1^)	122.0 (40.4)	72.0	265.0
Bicarbonate (mEq/dL^−1^)	25.9 (4.9)	16.5	30.9
Sodium (mEq/dL^−1^)	139.0 (5.9)	115.0	158.0
Potassium (mEq/dL^−1^)	4.18 (0.52)	2.54	5.41

IQR, interquartile range.

The most common treatments were medium-potency topical steroids (*n* = 38; 46%) and systemic steroids (*n* = 50), with doses ranging from 20 to 50 mg. Hospital stay was prolonged (>10 days) in 58% (*n* = 48) of patients, with a median of 11 days. There was only one admission to the intensive care unit and one death (1% each).

### Prolonged vs. nonprolonged hospital stay

When comparing prolonged and nonprolonged hospital stays (Table 3), palmoplantar and genital mucosal involvement was significantly more frequent in prolonged stays; eosinophilia, liver enzyme abnormalities, the use of high-potency steroids and elevated C-reactive protein were also present, and those who required opioids or antibiotics during hospitalization tended to have longer stays. It is noteworthy that pemphigus foliaceus was more frequent among those who did not have a prolonged hospital stay. It is also important to note that six patients had genital mucosa involvement and these six had a prolonged hospital stay. A single case of hypotension was identified, and this patient also had a prolonged hospital stay.

**Table 3:** Patient characteristics according to prolonged or nonprolonged hospital stay

Characteristics	Hospital stay, *n* (%)		*P*-value^a^	PR (95% CI)
	Prolonged (*n* = 48)	Nonprolonged (*n* = 35)		
History variables				
Biologic sex				
Female	33 (69)	18 (51)	0.11	1
Male	15 (31)	17 (49)		0.72 (0.47–1.10)
DDP4 inhibitor				
Yes	3 (6)	7 (20)	0.06	1
No	45 (94)	28 (80)		2.05 (0.78–5.38)
Diuretics				
Yes	4 (8)	6 (17)	0.22	1
No	44 (92)	29 (83)		1.50 (0.68–3.29)
Antibiotics				
Yes	8 (17)	6 (17)	0.95	1
No	40 (83)	29 (83)		1.01 (0.61–1.66)
Anticonvulsants				
Yes	2 (4)	1 (3)	0.75	1
No	46 (96)	34 (97)		0.86 (0.37–1.96)
NSAIDs				
No	45 (94)	35 (100)	0.13	1
Yes	3 (6)	0 (0)		1.77 (1.46–2.15)
Opioids				
No	46 (96)	35 (100)	0.22	1
Yes	2 (4)	0 (0)		1.76 (1.45–2.12)
History of neoplasia				
No	45 (94)	33 (94)	0.92	1
Yes	3 (6)	2 (6)		1.04 (0.49–2.18)
Haematological malignancy				
No	47 (98)	34 (97)	0.82	1
Yes	1 (2)	1 (3)		0.86 (0.21–3.48)
Solid organ malignancy				
No	46 (96)	33 (94)	0.75	1
Yes	2 (4)	2 (6)		0.85 (0.31–2.32)
History of recent infection				
No	40 (83)	27 (77)	0.48	1
Yes	8 (17)	8 (23)		0.83 (0.49–1.42)
History of arterial hypertension				
No	20 (42)	12 (34)	0.49	1
Yes	28 (58)	23 (66)		0.87 (0.60–1.26)
History of diabetes mellitus				
No	33 (69)	19 (54)	0.17	1
Yes	15 (31)	16 (46)		0.76 (0.50–1.15)
History of hypothyroidism				
No	41 (85)	31 (89)	0.68	1
Yes	7 (15)	4 (11)		1.11 (0.68–1.82)
History of dyslipidaemia				
No	30 (63)	22 (63)	0.97	1
Yes	18 (38)	13 (37)		1.00 (0.68–1.47)
History of obesity				
No	32 (67)	27 (77)	0.29	1
Yes	16 (33)	8 (23)		1.22 (0.85–1.77)
History of neurological disease				
No	32 (67)	27 (77)	0.29	1
Yes	16 (33)	8 (23)		1.22 (0.85–1.77)
History of psychiatric illness				
No	37 (77)	26 (74)	0.76	1
Yes	11 (23)	9 (26)		0.93 (0.59–1.46)
History of autoimmune disease				
No	46 (96)	32 (91)	0.41	1
Yes	2 (4)	3 (9)		0.67 (0.22–2.01)
Clinical variables				
Bullous pemphigoid				
No	20 (42)	18 (51)	0.37	1
Yes	28 (58)	17 (49)		1.18 (0.81–1.72)
Pemphigus foliaceus				
No	44 (92)	26 (74)	**0**.**03**	1
Yes	4 (8)	9 (26)		0.48 (0.21–1.12)
Pemphigus vulgaris				
No	35 (73)	29 (83)	0.28	1
Yes	13 (27)	6 (17)		1.25 (0.85–1.82)
IgA pemphigus				
No	47 (98)	34 (97)	0.82	1
Yes	1 (2)	1 (3)		0.86 (0.21–3.48)
Acquired epidermolysis bullosa				
No	47 (98)	35 (100)	0.39	1
Yes	1 (2)	0 (0)		1.74 (1.44–2.10)
Linear IgA dermatosis				
No	47 (98)	34 (97)	0.82	1
Yes	1 (2)	1 (3)		0.86 (0.21–3.48)
Percentage of body surface area				
<30%	25 (52)	22 (63)	0.29	1
>60%	4 (8)	2 (6)		1.25 (0.67–2.34)
30–60%	18 (38)	8 (23)		1.30 (0.89–1.88)
No data	1 (2)	3 (9)		0.47 (0.08–2.62)
Weals				
No	33 (69)	30 (86)	0.07	1
Yes	15 (31)	5 (14)		1.43 (1.01–2.02)
Eczematous lesions				
No	29 (60)	21 (60)	0.96	1
Yes	19 (40)	14 (40)		0.99 (0.68–1.44)
Desquamation				
No	26 (54)	21 (60)	0.59	1
Yes	22 (46)	14 (40)		1.10 (0.76–1.59)
Scalp involvement				
No	35 (73)	27 (77)	0.66	1
Yes	13 (27)	8 (23)		1.09 (0.73–1.63)
Oral mucosa involvement				
No	33 (69)	27 (77)	0.39	1
Yes	15 (31)	8 (23)		1.18 (0.81–1.72)
Conjunctiva involvement				
No	45 (94)	35 (100)	0.13	1
Yes	3 (6)	0 (0)		1.77 (1.46–2.15)
Genital mucosa involvement				
No	42 (88)	35 (100)	**0**.**03**	1
Yes	6 (13)	0 (0)		1.83 (1.49–2.24)
Palmoplantar involvement				
No	38 (79)	33 (94)	**0.05**	1
Yes	10 (21)	2 (6)		1.55 (1.11–2.17)
Impetiginization				
No	43 (90)	34 (97)	0.19	1
Yes	5 (10)	1 (3)		1.49 (0.99–2.24)
Hypotension				
No	47 (98)	35 (100)	0.39	1
Yes	1 (2)	0 (0)		1.74 (1.44–2.10)
Laboratory variables				
Haemoglobin				
No anaemia	33 (69)	22 (63)	0.46	1
Anaemia	15 (31)	12 (34)		0.92 (0.62–1.38)
White blood cell count				
Leucopenia	3 (6)	1 (3)	0.485	1
Normal	13 (27)	7 (20)		0.86 (0.45–1.66)
Leucocytosis	32 (67)	26 (74)		0.73 (0.39–1.35)
Platelet count				
Thrombocytopenia	1 (2)	1 (3)	0.751	1
Normal	38 (79)	30 (86)		1.11 (0.27–4.54)
Thrombocytosis	8 (17)	3 (9)		1.45 (0.34–6.09)
Eosinophil count				
Normal	26 (54)	23 (66)	**0**.**006**	1
Eosinophilia	21 (44)	6 (17)		1.46 (1.05–2.04)
Alanine transferase				
Normal	43 (90)	23 (66)	**0**.**03**	1
Elevated	1 (2)	3 (9)		0.38 (0.06–2.11)
Aspartate transferase				
Normal	41 (85)	25 (71)	**0**.**03**	1
Elevated	4 (8)	1 (3)		1.28 (0.79–2.07)
Gamma glutaryl transferase				
Normal	2 (4)	7 (20)	**0**.**02**	1
Elevated	5 (10)	0 (0)		4.5 (1.32–15.2)
Alkaline phosphatase				
Normal	23 (48)	5 (14)	**0**.**001**	1
Elevated	3 (6)	0 (0)		1.21 (1.02–1.44)
Urea				
Normal	25 (52)	16 (46)	0.83	1
Elevated	9 (19)	7 (20)		0.92 (0.56–1.51)
Creatinine				
Normal	38 (79)	18 (51)	**0**.**02**	1
Elevated	10 (21)	15 (43)		0.58 (0.35–0.98)
Lactate dehydrogenase				
Normal	8 (17)	4 (11)	0.13	1
Elevated	7 (15)	1 (3)		1.31 (0.81–2.11)
Glycaemia				
Normal	9 (19)	3 (9)	**0.07**	1
Hyperglycaemia	18 (38)	8 (23)		0.92 (0.60–1.39)
Bicarbonate				
Diminished	1 (2)	2 (6)	0.47	1
Normal	1 (2)	0 (0)		3 (0.60–14.8)
Elevated	4 (8)	1 (3)		2.4 (0.45–12.6)
Serum sodium				
Hyponatraemia	4 (8)	2 (6)	0.09	1
Normal	31 (65)	14 (40)		1.03 (0.56–1.88)
Hypernatraemia	1 (2)	2 (6)		0.5 (0.09–2.72)
Serum potassium				
Hypokalaemia	4 (8)	1 (3)	0.32	1
Normal	29 (60)	17 (49)		0.78 (0.48–1.28)
Hyperkalaemia	3 (6)	2 (6)		0.75 (0.32–1.73)
Treatment variables				
Topical steroid				
High potency	27 (56)	7 (20)	**0**.**005**	1
Low potency	0 (0)	2 (6)		IND^b^
Medium potency	19 (40)	19 (54)		0.62 (0.43–0.90)
Not received	1 (2)	5 (14)		0.20 (0.03–1.26)
In-hospital opioids				
No	33 (69)	32 (91)	**0.03**	1
Yes	14 (29)	2 (6)		**1.72 (1.27–2.33)**
In-hospital antibiotics				
No	25 (52)	29 (83)	**0.01**	1
Yes	22 (46)	5 (14)		**1.76 (1.25–2.47)**

CI, confidence interval; DPP4, dipeptidyl peptidase-4; IND, indeterminable; NSAID, nonsteroidal anti-inflammatory drug; PR, prevalence ratio.

a Stastistically significant values in bold.

b Indeterminate with this values.

### Multivariate analysis

Those who did not use DPP4 inhibitors showed a significant association with a higher probability of prolonged stay [adjusted prevalence ratio (aPR) 8.56, 95% CI 1.46–50.26; *P* = 0.02]. Eosinophilia was also significantly associated (aPR 5.40, 95% CI 1.28–22.65; *P* = 0.02) with a prolonged hospital stay (Table 4). In-hospital antibiotic use remained an associated factor (aPR 3.75, 95% CI 1.01–13.95; *P* = 0.05). However, although variables such as opioid use and palmoplantar involvement were significant in the bivariate analysis, they did not maintain adjusted significance. For this reason, the factors associated with the highest probability of stay were not using previous DPP4, presence of eosinophilia and receiving hospital antibiotics. Variables not previously mentioned did not maintain adjusted significance.

**Table 4:** Multivariate analysis

Characteristics	*P*-value^a^	PR (95% CI)	*P*-value^b^	aPR (95% CI)
Biologic sex				
Female	0.11	1	0.92	1
Male		0.72 (0.47–1.10)		0.94 (0.30–2.97)
DDP4 inhibitor				
Yes	0.06	1	**0.02**	1
No		2.05 (0.78–5.38)		8.56 (1.46–50.26)
Palmoplantar involvement				
No	**0.05**	1	0.45	1
Yes		1.55 (1.11–2.17)		2.02 (0.33–12.44)
Eosinophil count				
Normal	**0006**	1	**0.02**	1
Eosinophilia		1.46 (1.05–2.04)		5.40 (1.28–22.65)
In-hospital opioids				
No	**0.03**	1	0.21	1
Yes		1.72 (1.27–2.33)		3.06 (0.53–17.5)
In-hospital antibiotics				
No	**0.01**	1	**0.05**	1
Yes		1.76 (1.25–2.47)		3.75 (1012–13.95)

Bold denotes statistical significance. aPR, adjusted prevalence ratio; CI, confidence interval; DPP4, dipeptidyl peptidase-4 ; PR, prevalence ratio.

a Original bivariate *P-*value.

b Adjusted multivariate *P-*value.

### Profile analysis

Within the profile analysis, patients with a nonprolonged hospital stay tended to be men, did not present genital mucosa and palmoplantar involvement, had used DPP4 inhibitors and did not have eosinophilia. Patients with prolonged stays were women, had normal and elevated alkaline phosphatase, in addition to eosinophilia, hypotension and palmoplantar involvement, and not having alanine transferase elevations.

## Discussion

AIBDs accounted for 2.8% of dermatology consultations (*n* = 83/3000). In general, these diseases are infrequent, with pemphigus vulgaris and pemphigoid being the main ones, with a prevalence of between 0.38 and 30 cases per 100 000 people per year for the former and 1.46 to 47.99 cases per 100 000 for the latter.^11–15^ In a cross-sectional study from Khartoum, pemphigus vulgaris was the most common autoimmune blistering disorder (32.6%) and BP the second most common (29.3%), with a female predominance (58.7%); by contrast, in our cohort, BP was seen most frequently (54.2%), while the female predominance was concordant (61.4%).^16^ The number of cases reported is comparable to that reported by Camacho-Pérez *et al*., where 21 patients with pemphigus were evaluated over a 5-year period, and Zuluaga *et al*., with 99 cases of pemphigus and pemphigoid over 16 years.^17,18^

Although no hospital-stay studies have been carried out for AIBDs as a group, Ren *et al*. reported an average hospital stay of 7.3 days for BP in addition to a mortality rate of 2.3%, comparable to the median of 11 days and mortality rate of 1.2% reported in the present study, even including other bullous pathologies such as pemphigus foliaceus which, due to its smaller spread, limitation to seborrhoeic areas and absence of mucosal involvement, is associated with shorter stays.^3^ Additionally, it was clearly observed that there is marked metabolic and cardiovascular comorbidity due to hypertension, diabetes and dyslipidaemia, with results similar to those reported by Sánchez-García *et al*., as well as other studies that evaluated comorbidity.^19,20^ While this can be partly explained by the presentation of these diseases in older individuals, it also implies that they are patients at higher risk of a major cardiovascular event.^21,22^

The association of the use of DPP4 inhibitors with shorter hospital stays is also highlighted. In the last 10 years, the relationship between the use of this group of drugs and the development of BP has been identified.^23,24^ Considering that BP is associated with longer hospital stays and refractoriness in treatment, this association can be interpreted as meaning that in patients with an associated drug trigger, withdrawal of this drug implies a faster response to hospital management.

In contrast to the most relevant clinical characteristics, the involvement of the genital mucosa and palmoplantar involvement stands out. Although these areas may not represent large body surfaces, they imply areas of great morbidity and limitation, which may explain the prolongation of the hospital stay.^25–29^ In a retrospective study by Garrido *et al*., a relationship was found between elevated serum eosinophil levels and palmoplantar involvement, which may partly support the association of involvement in this anatomical area with prolonged hospital stay.^30^

Regarding the paraclinical findings associated with prolonged hospital stay, the importance of eosinophilia should be highlighted due to its sustained significance in the multivariate analysis. Given that more than half of patients in our study were diagnosed with BP, it is coherent with the predominance of eosinophilia. Consistent with the results of a prior retrospective cohort study, the requirement for inpatient care was significantly associated with higher baseline peripheral blood eosinophil counts, and relapse was more frequent among patients presenting with higher eosinophil levels. Peripheral eosinophil counts also correlated with circulating anti-BP180 IgG and IgE titres and with overall disease severity, supporting the biologic plausibility of an eosinophil-driven, type-2 inflammatory endotype in BP.^30–33^ Although the correlation between these specific variables was not assessed, they could correspond to the reason behind the need to extend the stay.

Among the other laboratory variables, liver enzymes of both hepatocellular and cholestatic patterns stand out, as does the elevation of C-reactive protein. Regarding liver enzymes, the literature reports elevations associated with the use of immunosuppressive treatment, as well as a case of paraneoplastic pemphigus secondary to hepatocarcinoma.^34,35^ None of these situations would explain the elevations in the cases presented and as such they could represent a sign of comorbidity or even damage associated with the same pathology, although there are no current data to support this theory. However, an elevation in C-reactive protein has been correlated with the activity of bullous disease, reflecting the underlying inflammatory process, and can be a confounding factor in the face of an underlying infectious process, so it must be evaluated over time.^36–39^

Elhag *et al*. conducted a cross-sectional study at the Khartoum Dermatology and Venereal Diseases Hospital, providing supportive evidence of the positive role of complement components, particularly C3, in diagnosing pemphigus, with a good correlation with disease severity. As our study did not include IgG or complement levels among the variables analysed, we did not perform an exhaustive search for an association between these variables and prolonged hospital stay. However, these markers could prove useful in assessing the prognosis of patients with AIBDs.^16^

The use of hospital antibiotics is highlighted as a factor associated with prolonged stay. Patients with blistering diseases, having a compromised skin barrier, are more susceptible to developing infections in both the ambulatory and nosocomial settings.^3,6,22,40^ A retrospective study of hospitalized patients with pemphigus at Peking University First Hospital found that those with bacterial skin infections had a significantly longer duration of stay than those without infections (median 18.9 vs. 14.1 days).^41^ Careful management of isolation and protection measures can impact the development of these infections and prolong stays due to the need for antibiotic use.

Systemic corticosteroids are the cornerstone of treatment for severe blistering diseases, including in hospitalized patients. However, when steroid monotherapy fails to achieve adequate disease control or is contraindicated because of patient comorbidities, adjunctive therapies can be added, such as steroid-­sparing agents, antibiotics, ciclosporin, mycophenolate mofetil, rituximab and intravenous immunoglobulin. Nonetheless, the disease in a subset of patients remains refractory or patients experience significant adverse effects, which has driven the development of targeted therapies based on a more advanced understanding of the pathophysiology of these conditions. Emerging options include dupilumab, omalizumab, anti-eosinophil strategies, complement-directed therapies, Janus kinase inhibitors and FcRn antagonists, which have shown effectiveness and offer a path towards reducing the toxicity associated with broad immunosuppressive regimens; however, although studies are underway, there is still no published evidence specifically documenting their use in hospitalized patients, and well-designed trials are needed to evaluate their efficacy and safety in these settings.^42,43^

The main limitation of this retrospective study is the use of a secondary source for data collection, which can introduce information bias due to the lack of homogeneity in the recording of clinical and paraclinical parameters. Additionally, not all patients had the same laboratory tests available, which could limit the generalizability of certain findings. However, laboratory and pathology results were retrieved directly from the institutional system, minimizing the risk of interpretive bias from treating physicians. This bias was also reduced through standardization in the ­data-collection process during a pilot test. Taking this into account, this study continues to have external validity as it represents a census of the evaluated population, which can be extrapolated to other institutions with hospital dermatology services.

The present study highlights how most patients with AIBDs bullous diseases have prolonged hospital stays, which implies higher costs and increased risk of infection and adverse outcomes. The evaluation of triggering factors, such as the use of DPP4 inhibitors, for early discontinuation may impact this outcome. In addition, the importance of evaluating the mucous membranes and the palmoplantar region as possible predictors of prolonged stay is highlighted. In addition to this, basic investigations that include a blood count, liver enzymes and inflammatory markers are undertaken to identify patients who are most likely to have prolonged stays. Finally, cautious biosecurity measures must be taken to avoid infections that negatively impact the hospital stay, and morbidity and mortality of these patients. In the future, we suggest conducting prospective studies in which clinimetric assessments, laboratory tests and histopathological reporting are standardized. This would better establish causality and the predictive role of these factors in prolonged hospital stays.
